# Gaining power and precision by using model–based weights in the analysis of late stage cancer trials with substantial treatment switching

**DOI:** 10.1002/sim.6801

**Published:** 2015-11-17

**Authors:** Jack Bowden, Shaun Seaman, Xin Huang, Ian R White

**Affiliations:** ^1^ MRC Integrative Epidemiology UnitUniversity of BristolBristolU.K.; ^2^ MRC Biostatistics UnitCambridgeU.K.; ^3^ PfizerLa JollaSan DiegoCAU.S.A.

**Keywords:** late‐stage cancer, logrank test, RPSFTM, treatment switching

## Abstract

In randomised controlled trials of treatments for late‐stage cancer, it is common for control arm patients to receive the experimental treatment around the point of disease progression. This treatment switching can dilute the estimated treatment effect on overall survival and impact the assessment of a treatment's benefit on health economic evaluations. The rank‐preserving structural failure time model of Robins and Tsiatis (*Comm. Stat.*, **20**:2609–2631) offers a potential solution to this problem and is typically implemented using the logrank test. However, in the presence of substantial switching, this test can have low power because the hazard ratio is not constant over time. Schoenfeld (*Biometrika*, **68**:316–319) showed that when the hazard ratio is not constant, weighted versions of the logrank test become optimal. We present a weighted logrank test statistic for the late stage cancer trial context given the treatment switching pattern and working assumptions about the underlying hazard function in the population. Simulations suggest that the weighted approach can lead to large efficiency gains in either an intention‐to‐treat or a causal rank‐preserving structural failure time model analysis compared with the unweighted approach. Furthermore, violation of the working assumptions used in the derivation of the weights only affects the efficiency of the estimates and does not induce bias or inflate the type I error rate. The weighted logrank test statistic should therefore be considered for use as part of a careful secondary, exploratory analysis of trial data affected by substantial treatment switching. ©©2015 The Authors. *Statistics inMedicine* Published by John Wiley & Sons Ltd.

## 1 Introduction

In randomised controlled trials (RCTs) of late‐stage cancer therapies, it is common to give the experimental treatment to placebo arm patients at the point of disease progression. This could occur for several reasons: an individual clinician may feel it the best course of action for their patient; it may be pre‐specified in the trial protocol as part a dynamic treatment strategy; or emerging evidence (internally or externally to the specific RCT) of the active treatment's benefit may have broken the original trial equipoise. Regardless of the reason, treatment switching (also called contamination or cross‐over) does not generally affect the assessment of early outcome measures such as progression free survival but can substantially dilute the estimated treatment effect on overall survival. For example, Demetri *et al.*, 1 reported the results of a randomised controlled trial into the use of Sunitinib for the treatment of advanced gastrointestinal stromal tumours in patients for whom conventional therapy (Imatinib) had failed because of resistance or intolerance. Early trial results were unequivocal; randomisation of patients to either placebo or Sunitinib stopped early after a planned interim analysis showed a strong benefit in favour of the new treatment in terms of time to tumour progression (hazard ratio 0.33, *p*< 0.001). However, subsequent analysis and interpretation of the data were made harder by the understandable decision to change to an open label protocol and make Sunitinib available to the patients in the placebo arm, which led to the vast majority of eligible patients in the placebo arm switching to receive Sunitinib. By the end of follow‐up, the intention‐to‐treat (ITT) hazard ratio between treatment and control groups for overall survival — the original primary outcome — had weakened from 0.49 (*p*= 0.007) at the interim analysis to 0.88 (*p*= 0.31).

Another example of this phenomenon is described by Motzer *et al.*, 2, who reported the results of a double blind Phase III randomised trial into the use of Everolimus for patients with advanced renal cell carcinoma. In order to address ethical concerns and improve recruitment rates, the trial protocol stipulated that, upon disease progression or unacceptable toxicity, patients were to be unblinded and given the option to switch to an open‐label Everolimus. After 8months of follow up, the Everolimus arm patients were experiencing significantly fewer disease progressions (progression–free survival hazard ratio 0.3, *p*< 0.001) but after 10months, any early difference in overall survival had disappeared (OS hazard ratio 0.83, *p*= 0.23).

In both cases, a strong and early treatment benefit in progression free survival was enough for licence approval by industry regulators. So why, one may ask, does the subsequent artificial dilution in the estimate of overall survival matter? One reason is that it makes it far harder for authorities, such as the UK's National Institute of Clinical Exellence (NICE), to subsequently assess whether these treatments are cost‐effective. This is because their calculations rely heavily on an accurate, unbiased measure of overall survival, which trials of this nature do not readily provide 3.

This issue does not just affect industry sponsored trials that seek to gain approval for new treatments. The Concorde trial 4 evaluated the relative effectiveness of two pragmatic approaches to the management of patients with HIV with a proven therapy (Zidovudine): one arm (the Imm group) received Zidovudine immediately, whilst the other arm — the deferred (or Def) group — received it when their condition deteriorated to AIDS Related Complex (ARC). However, whilst the study was ongoing, a change was made to the protocol to allow those in the Def arm to receive Zidovudine if they experienced a persistently low CD4 count, in order to be in line with current clinical practise of the time. At the end of the trial, only a small non‐significant difference could be detected between the two arms in terms of overall survival. Several authors subsequently queried whether a larger difference would have been observed if the original protocol had been followed (see 5 for example).

The rank preserving structural failure time model (RPSFTM) of Robins and Tsiatis 6 can be used to estimate the causal effect of a randomised treatment allowing for contamination. The method is based on the initial randomisation of patients and requires specification of an ITT test (typically, the logrank test). The procedure for testing the null hypothesis of no causal effect of treatment in the RPSFTM is *exactly* the same as testing for no effect of treatment assignment in an ITT analysis. *P*‐values resulting from the RPSFTM and ITT analyses are consequently identical. For this reason, and also because it does not require an assumption of no unmeasured confounders, the RPSFTM has been acknowledged by NICE as the most statistically principled and robust analysis procedure in this setting 7. It has been applied to the above trials in subsequent analyses, for example, in the case of the Concorde trial by White *et al*. 8. Unfortunately, the logrank test can suffer from a loss of power — so that one consistently fails to reject the ITT null hypothesis even when the treatment is strongly effective — if a substantial portion of the participants switch treatments during the course of the trial. This is because the logrank test is optimal when the hazard ratio is constant, whilst treatment switching causes the hazard ratio to change over time (when the treatment effect is non‐zero and constant). Our aim in this paper is to find methods of analysis that adjust for treatment switches whilst respecting the randomisation, but that give greater power to the ITT test and consequently greater precision to the causal effect estimator than the standard implementation of the RPSFTM. This is achieved by building on the work of Schoenfeld 9 and Lagakos *et al*. 10, who developed weighted versions of the logrank ITT test. The weights in our logrank ITT test are largely driven by the proportion of people on the active treatment in each arm over time, which we feel is simple and heuristically appealing.

In Section 2, we review the standard and weighted forms of the logrank test and use working assumptions to derive approximately ‘optimal’ weights for testing the ITT null hypothesis using the weighted logrank test. In Section 3, we review the RPSFTM framework in 6 and discuss how it can be implemented using our weighted logrank test approaches. In Section 4, we use Monte‐Carlo simulation to compare the performance of the standard and weighted logrank tests when used for hypothesis testing within an ITT analysis (in terms of power and type–I error) and within an RPSFTM analysis (in terms of bias and mean‐squared error of estimates for the causal effect parameter). In Section 5, we apply our new methodology to a re‐analysis of the Sunitinib and Concorde trials. We conclude in Section 6 with a discussion of the issues raised and point to future research.

## 2 Logrank tests for intention‐to‐treat analysis

### 2.1 The standard and weighted logrank tests

Consider a clinical trial assessing whether a new ‘experimental’ treatment is superior to standard (control) therapy. Let *T*_1_,…,*T*_*n*_ denote the failure or censoring times of *n* participants randomised into the trial. Let $T_{\left(1\right)}\leq \ldots \leq T_{\left(J\right)}$( J≤n) denote the *J* ordered observed event times. The logrank test statistic for the null hypothesis, *H*_0_, that there is no difference between the distribution of survival times in the experimental treatment and control arms can be written as follows:

(1) $$Z=\frac{\overset{J}{\underset{j=1}{\sum }}\left(O_{j}-E_{j}\right)}{\sqrt{\overset{J}{\underset{j=1}{\sum }}V_{j}}}.$$

*O*_*j*_ is the observed number of failures in the treatment arm at time *T*_(*j*)_. *E*_*j*_ is the expected number of failures at time *T*_(*j*)_ in the treatment arm given the total number of failures at time *T*_(*j*)_, and *V*_*j*_ is its variance, both under *H*_0_.

The weighted logrank test statistic is

(2) $$Z^{W}=\frac{\overset{J}{\underset{j=1}{\sum }}W_{j}\left(O_{j}-E_{j}\right)}{\sqrt{\overset{J}{\underset{j=1}{\sum }}W_{j}^{2}V_{j}}},$$

where *W*_1_,…,*W*_*J*_ are a set of weights. Schoenfeld 9 derived the optimal weights for the logrank test, *W*1opt,…,*W* *J*opt, that is. the weights that asymptotically maximise its power to reject *H*_0_ under the alternative hypothesis. He showed that $W_{\left(j\right)}^{\text{opt}}$ is proportional to the true log hazard ratio at time *T*_(*j*)_. Hence, if the true log hazard ratio is constant, the standard (unweighted) logrank test is optimal. However, when patients are permitted to switch treatment over the course of follow‐up, the log hazard ratio will, in general, not be constant, and so a weighted logrank test will be optimal.

Lagakos *et al*. 10 explored the relative efficiency of the weighted logrank test using the optimal weights compared with the unweighted logrank test in a two‐arm RCT in which there is non‐compliance in the treatment arm but perfect compliance in the control arm. They assumed that censoring times were independent of both failure times and treatment switching times, as would be the case if censoring were administrative. In the next section, we modify their approach to derive optimal weights in the general situation where treatment switching can occur in the treatment and control arms. We continue to make the working assumption that censoring is independent of failure and treatment switching.

### 2.2 An ‘optimal’ weighted logrank intention‐to‐treat test

Let *X*_*i*_(*t*) be a binary random variable with *X*_*i*_(*t*)= 1 if person *i* is on the experimental treatment at time *t*; *X*_*i*_(*t*)= 0 if they are off the experimental treatment (and therefore on the control treatment), and let *x*_*i*_(*t*) be its observed value. We assume throughout that it is only possible, at each time point, to take the experimental treatment/control treatment fully or not at all (although this could be relaxed easily). We write ${\bar{x}}_{i}(t)=\{x_{i}(s):0\leq s\leq t\}$ to represent person *i*'s full treatment history up to time *t*. Further, let *R*_*i*_=*r* be their randomisation indicator so that, at *t*= 0, subject *i* receives either the experimental treatment (if *R*_*i*_= 1) or control (if *R*_*i*_= 0), and *x*_*i*_(0) = *R*_*i*_. For *t*∈(0,*T*_*i*_], a person's treatment may, however, depart from the original assignment so that *x*_*i*_(*t*) is not necessarily equal to *R*_*i*_. The reason for this departure may be associated with the underlying health of the patient.

Suppressing the subscript *i* for convenience, let

$$\;h^{r}(t)=h(t|R=r)=\lim_{\Delta \to 0}\;\frac{P(T\leq t+\Delta |T\geq t,R=r)}{\Delta }$$

denote the conditional hazard of failure at time *t* given randomisation to arm *r*. Let $h_{\text{on}}^{r}(t)$ = *h* ^*r*^(*t*|*x*(*t*) = 1) and $h_{\text{off}}^{r}(t)$ = *h* ^*r*^(*t*|*x*(*t*) = 0) denote the conditional hazards of failure at time *t* given randomisation to arm *r* and given that at time *t* treatment is, respectively, being received and not being received. Note that these hazards depend not only on the distribution of failure times in the absence of treatment and on the true treatment effect but also on the treatment switching process. In order to derive optimal weights, we make the following ‘working’ assumptions. It is important to first stress that WA1 and WA2 are not necessary for the weighted logrank test asymptotically to maintain its nominal type‐I error rate. Only the non‐informative censoring assumption is required, and this is also required by the standard (unweighted) logrank test.

- Working assumption 1 (WA1): $h_{\text{off}}^{0}(t)=h_{\text{off}}^{1}(t)$ and $h_{\text{on}}^{0}(t)=h_{\text{on}}^{1}(t)$.

Given WA1, we can ignore the randomisation superscript and denote the hazard functions simply as *h*_on_(*t*) and *h*_off_(*t*).

- Working assumption 2 (WA2): $\log\{h_{\text{on}}(t)/h_{\text{off}}(t)\}=\theta_{0}\;\forall t$.

In a slight modification of the words used by Lagakos *et al*., these assumptions would hold if, at any given time, patients switch treatment randomly and independently of their underlying health at that time. We stress that this is an implausible assumption which is not required for the validity of the analysis. Let *γ* ^*r*^(*t*) = $\mathrm{Pr}(X_{i}(t)=1|T\geq t,R_{i}=r)$ denote the conditional probability of being on treatment at time *t* given randomisation to arm *r* and given that failure has not occurred prior to time *t*. Then, using WA1

$$\;h^{r}(t)=\gamma^{r}(t)h_{\text{on}}(t)+\left\{1-\gamma^{r}(t)\right\}h_{\text{off}}(t)$$

and so using WA2, the log hazard ratio at time *t* comparing the two randomisation arms is

$$\log\left(\frac{h^{1}(t)}{h^{0}(t)}\right)=\log\left(\frac{1+\gamma^{1}(t)e^{\theta_{0}}-\gamma^{1}(t)}{1+\gamma^{0}(t)e^{\theta_{0}}-\gamma^{0}(t)}\right).$$

Therefore, under our working assumptions, the optimal weight for the *j*th failure is

(3) $$W_{j}^{\text{opt}}=\log\left\{1+\left[\gamma^{1}\left(t_{\left(\;j\right)}\right)\right]\left(e^{\theta_{0}}-1\right)\right\}-\log\left\{1+\left[\gamma^{0}\left(t_{\left(\;j\right)}\right)\right](e^{\theta_{0}}-1)\right\}$$

If, moreover, *θ*_0_ is close to, but not equal to, zero, we can use the fact that log(1+x)≈x for small *x* to derive from equation (3) that

$$W_{j}^{\text{opt}}\approx \left\{\gamma^{1}\left(t_{\left(\;j\right)}\right)-\gamma^{0}\left(t_{\left(\;j\right)}\right)\right\}(e^{\theta_{0}}-1)$$

The $\left(e^{\theta_{0}}-1\right)$ term cancels in Equation (2) and so we can write

(4) $$W_{j}^{\text{opt}}\approx \gamma^{1}\left(t_{\left(\;j\right)}\right)-\gamma^{0}\left(t_{\left(\;j\right)}\right).$$

We will refer to the weights defined in Equation (4) as ‘simple ITT weights’. If WA1 and WA2 are violated, they could lose some of their efficiency to detect a treatment effect when one truly exists. Of course, there may be very good reasons to doubt the validity of WA1 and WA2 in the late‐stage cancer context, when treatment switching is often initiated by the progression of a patient's disease. We address this issue in detail in Section 4 and in the Discussion.

### 2.3 Is it acceptable to weight by treatment usage in an intention‐to‐treat analysis?

In this paper, we follow Lagakos *et al*. 10 in referring to our approach as a weighted ITT test. We do so because it adheres to the ITT principles of basing the analysis on a comparison between the original randomised groupings and including all patients in the analysis. However, some may disapprove of our use of this terminology. Indeed, a reviewer has expressed serious concern that, whilst ITT tests are perfectly entitled to weight patients differently (for example, the Fleming‐Harrington family of tests 11, 12 allows the weight given to the *j*th patient's failure to be a pre‐specified function of the survival proportion at time *t*_(*j*)_ ) treatment usage rates should *not* be used for this purpose. We understand the reviewer's concerns and stress that this weight is not a function of patient *j*'s individual treatment usage, as in a *per‐protocol* or *as treated* analysis say but is based on the treatment usage of the entire study population at time *t*_(*j*)_. Of course, it would be unacceptable if the type I error rate of the weighted logrank test was inflated because of the estimation of *γ* ^0^(*t*) and *γ* ^1^(*t*). Lagakos *et al*. noted that in the simpler scenario, they considered, where there was non‐compliance only in one arm, the nominal type‐I error rate was achieved (despite estimation of their weights). Using simulation studies, we shall investigate whether this is also true in our setting. It would also be a concern if the weighted test encouraged erroneous interpretations of the data under the alternative hypothesis. We shall return to this important issue within the context of a hypothetical scenario in the Discussion section.

## 3 Logrank tests for causal inference

### 3.1 The rank‐preserving structural failure time model

If a substantial proportion of patients depart from their originally allocated treatment, then an ITT analysis, which would provide an estimate of the causal effect of treatment *assignment*, could differ substantially from the causal effect of the treatment itself. The RPSFTM provides a statistical framework for estimating this causal effect, by linking each patient's event time (*T*) to their (possibly counterfactual) event time had they had not received any treatment (*T*_0_) via the formula:

(5) $$T_{0}=T_{0}(\beta )=\overset{T}{\underset{0}{\int }}\exp\left\{\mathrm{\beta x}(t)\right\}\mathrm{dt}$$

The single parameter *β*, indexing the RPSFTM, can be interpreted in the following manner. The rate at which a person's lifetime is used up is *e* ^*β*^ times greater when on treatment than when off treatment. For example, if β=log(0.5)≈‐0.7 then treatment slows this rate by a half. If *β* = 0, then *T*_0_=*T*, meaning that treatment has no causal effect on survival time. If we write *T* as *T* = *T*_on_+*T*_off_, where *T*_on_ and *T*_off_ denote times spent on and off treatment, respectively, then (5) can be written as *T*_0_(*β*) = *e* ^*β*^ *T*_on_+*T*_off_. We shall refer to the *T*_0_(*β*)'s and any statistic that is a function of them as being on the ‘*β*‐transformed’ timescale and denote the true value of the causal parameter *β* as *β*_0_.

In order to estimate *β*_0_, we use the fact that, under the RPSFTM, *T*_0_(*β*_0_) is independent of *R*. For each value of *β* in a range of possible values, the null hypothesis *H*_0,*β*_ that *T*_0_(*β*)⊥⊥*R* is tested using an appropriate test statistic. We first consider the commonly used logrank test statistic (and variations thereof). If *Z*(*β*) denotes the logrank test statistic calculated using Equation (1) with the *β*‐transformed failure times, then *E*{*Z*(*β*)}=0 when *T*_0_(*β*)⊥⊥*R*. So *β*_0_ can be estimated as the value $\hat{\beta }$ of *β* for which $Z(\hat{\beta })$ crosses zero (that is, where sign( $Z(\hat{\beta }-\varepsilon )$) ≠ sign( $Z(\hat{\beta }+\varepsilon )$) for some small value of *ε*). This method is often referred to as ‘g–estimation’. A 100(1‐*α*)% confidence interval for *β*_0_ can be obtained by finding the range of values of *β* for which $|Z(\beta )|\leq z_{1-\alpha /2}$. This is the set of values for which *H*_0,*β*_ can not be rejected at significance level *α*. Note this is typically not symmetrical around $\hat{\beta }$.

### 3.2 Weighted logrank tests for the rank‐preserving structural failure time model

In this section, we consider the use of the weighted log‐rank test statistic in a RPSFTM analysis to estimate the causal effect of treatment in the presence of treatment switching. Intuitively, the idea is to apply the RPSFTM not only to the event time but also to the treatment change times, and hence to apply the ideas of Section 2.2 on the *T*_0_(*β*) scale. In particular, we derive, for any given *β*, optimal weights for the test of the null hypothesis that *T*_0_(*β*)⊥⊥*R* under the following assumptions:

- Basic causal assumption (BCA): RPSFTM (5) holds.
- Causal working assumption 1 (CWA1): Patients switch treatment at random independently of their treatment‐free failure times *T*_0_(*β*_0_).
- Causal working assumption 2 (CWA2): *T*_0_(*β*_0_) ∼ *e* *x* *p*(*λ*) for some rate parameter *λ*.

Assumption CWA1 differs from WA1 and WA2 because using the RPSFTM requires us to condition on the complete treatment history, not just on current treatment. Assumption CWA2 is used to make the RPSFTM yield a simple model for the hazard. BCA is needed for consistency of the g–estimator of *β* even when the unweighted log rank test statistic is used. CWA1 and CWA2 are only needed for optimality of the weights. Thus, if CWA1 and CWA2 are violated but BCA holds then, we still obtain a consistent estimate for *β*_0_.

Just as in Section 2.2, the optimal weights we derive are functions of the ratio of the hazard of failure in the treatment arm relative to the hazard in the control arm. As before, they depend on the proportion of patients in each arm that are on treatment at a given time. However, unlike in Section 2.2, the hazards of interest, here, are the hazards of the *β*‐transformed time *T*_0_(*β*) in the two arms, rather than those of the untransformed time *T*. This is because we are testing *T*_0_(*β*)⊥⊥*R* when fitting the RPSFTM, as opposed to testing the ITT null hypothesis that *T*⊥⊥*R*. We now derive the hazard for *T*_0_(*β*).

Let an individual's treatment switching times be denoted *S*_1_<*S*_2_<…<*S*_*K*_<*T*, where *K* denotes the number of switches before failure, and let *S*_0_=0. Let *X*_*k*_ denote his treatment (0 if off treatment and 1 if on treatment) during time interval [*S*_*k* − 1_,*S*_*k*_). In order to make things more concrete, we will illustrate the general setup by referring to the special case where *β*_0_ = $\log\frac{3}{4}$ and to a single hypothetical patient, Peter. For Peter,

$$K=3,(S_{1},S_{2},S_{3})=(1,2,3),T=4,X_{1}=X_{3}=0,X_{2}=X_{4}=1$$

(for convenience, we assume that time is measured in years). Peter starts the trial off treatment (at year *S*_0_ = 0) and proceeds to have three treatment switches (at the start of years 1,2 and 3, respectively) before failing at the start of year 4. According to the RPSFTM, Peter's treatment‐free lifetime is *T*_0_(*β*_0_) = *e* *x* *p*(*β*_0_)*T*_on_ + *T*_off_. Therefore, his treatment free lifetime *T*_0_(*β*_0_) is expended at a rate of 1year per year of observed (or untransformed) study time whilst he is off treatment, but at a rate of $\exp(\beta_{0})$ = 3/4 years per year of observed study time whilst he is on treatment. In general, a unit of untransformed time corresponds to either $\exp(\beta_{0})$ units or one unit of *β*_0_‐transformed time.

We derive the hazard of *T*_0_(*β*), in general, that is, for any switching pattern and any *β*_0_ and *β*, and then for the hypothetical individual, Peter, using *β*_0_ = $\log\frac{3}{4}$ (the true value) and $\beta =\log\frac{1}{2}$ (our guess at the true value). In order to do this, we first derive the hazard function $h^{r}\{t|\bar{x}(t)\}$ of *T* in the same (general, then specific) manner.

### 3.3 Step 1: Derivation of the hazard of *T*

Our assumptions state that the *β*_0_‐transformed failure time *T*_0_(*β*_0_) is independent of randomisation arm (BCA) and treatment switching times (CWA1) and has a constant hazard *λ* (CWA2). It follows that the hazard of *T* at time *t* given randomisation arm and treatment history up to time *t* depends only on treatment at time *t* and equals $\lambda \exp(\beta_{0})$ whilst on treatment and *λ* whilst off treatment, that is,

(6) $$\begin{array}{cc} h^{r}\{t|\bar{x}(t)\} & =h^{r}\{t|\bar{x}(t)\}\exp\{\beta_{0}x(t)\}\\ \text{which, given CWA1} & =h(t)\exp\{\beta_{0}x(t)\}\\ \text{and CWA2} & =\lambda \exp\{\beta_{0}x(t)\}. \end{array}$$

**Figure 1:**
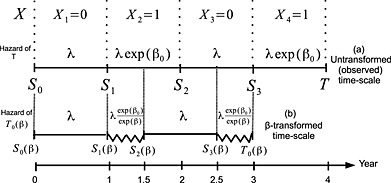
An illustration, for a hypothetical patient, Peter, of the relative switching times and corresponding hazard rates on: (a) the observed timescale and (b) the *β*‐transformed timescale.

This is illustrated in Figure 1(a) for Peter. His *T* is the outcome of a failure process in which the hazard is *λ* during the first year, $\lambda \exp(\beta_{0})=\frac{3\lambda }{4}$ during years 1–2, *λ* during years 2–3 and $\lambda \exp(\beta_{0})=\frac{3\lambda }{4}$ from years 3–4.

### 3.4 Step 2: Derivation of the hazard of *T*_0_(*β*)

We can now derive the hazard of the *β*‐transformed failure time *T*_0_(*β*) for any switching pattern and for any given *β*. When *T*_0_(*β*) is calculated from *T* using the RPSFTM (Equation (5)), each year of observed (untransformed) study time on treatment becomes exp(β) years of *β*‐transformed time. So the length of each period on treatment is multiplied by exp(β) and, crucially, the hazard of *T*_0_(*β*) during this period is divided by exp(β). The length of, and hazard during, periods off treatment remain unchanged. In general, we transform the *k*th switching time on the observed time scale, *S*_*k*_, to the switching time on the *β*‐transformed timescale, *S*_*k*_(*β*), via the formula

(7) $$S_{k}(\beta )=\overset{k}{\underset{j=1}{\sum }}\left(S_{j}-S_{j-1}\right)\exp\left\{\beta X_{j}\right\},$$

where *S*_0_(*β*) = 0. Using Equation (6), and dividing through by exp(β), the hazard of *T*_0_(*β*) is given by

(8) $$\lambda \exp\left\{(\beta_{0}-\beta )X_{k}\right\},$$

during interval [*S*_*k* − 1_(*β*),*S*_*k*_(*β*)). We now calculate the *β*‐transformed hazard for our hypothetical individual, Peter, using the specific value *β* = $\log\frac{1}{2}$. This is illustrated in Figure 1 (b). From Equation (7), *S*_*k*_(*β*), *k*=1,2,3 and *T*_0_(*β*) equal 1, 1.5, 2.5 and 3, respectively. For Peter, *T*_0_(*β*) is the outcome of a failure process in which the hazard is *λ* during the first year, $\lambda \exp(\beta_{0})/\exp(\beta )=\frac{3\lambda }{2}$ during years 1–1.5, *λ* during years 1.5–2.5 and $\frac{3\lambda }{2}$ during years 2.5–3. Note that, if *β* were *actually* equal to *β*_0_, then the hazard of *T*_0_(*β*) would equal a constant value of *λ*, in line with CWA2.

### 3.5 Step 3: Derivation of the optimal weights

We now derive the optimal weights for the RPSTM using the hazard of the *β*‐transformed failure time, *T*_0_(*β*). The argument is similar to that in Section 2.2. Let *x*(*t*;*β*) = *X*_*k*_ for *t*∈[*S*_*k* − 1_(*β*),*S*_*k*_(*β*)) represent the treatment indicator on the *β*–transformed timescale, and let $\bar{x}(t;\beta )=\{x(s;\beta ):0\leq s\leq t\}$. So the hazard of *T*_0_(*β*) at time *t* given randomisation arm *R*=*r* and *x*(*t*;*β*), that is, $\lim_{\mathrm{\Delta t}\to 0}P\{t\leq T_{0}(\beta )<t+\mathrm{\Delta t}|\bar{x}(t;\beta ),R=r,T_{0}(\beta )\geq t\}/\mathrm{\Delta t}$, is equal to $\lambda \exp\{(\beta_{0}-\beta )x(t;\beta )\}$ from Equation (8). It follows that the hazard of *T*_0_(*β*) at time t given *R* = *r* equals $\lambda \exp\{(\beta_{0}-\beta )\}\gamma^{r}(t;\;\beta )+\lambda \{1-\gamma^{r}(t;\;\beta )\}$, where $\gamma^{r}(t;\;\beta )=P(x(t;\beta )=1|T_{0}(\beta )\geq t,R=r)$.

Let *t*_(*j*),*β*_ denote the *j*th of the ordered *β*‐transformed failure times *T*_0_(*β*) (*j* = 1,…,*J*). Straightforward application of the results of Section 2.2 shows that the optimal weights for the weighted log rank test of the null hypothesis that *T*_0_(*β*)⊥⊥*R* are given by

(9) $$\begin{array}{cc} W_{j}^{\text{opt}}(\beta )= & \log\left\{1+\gamma^{1}\left(t_{(\;j),\beta };\beta \right)\left(e^{\beta_{0}-\beta }-1\right)\right\}\\ -\log\left\{1+\gamma^{0}\left(t_{(\;j),\beta };\beta \right)(e^{\beta_{0}-\beta }-1)\right\}, \end{array}$$

(*j* = 1,…,*J*). Moreover, if *β* is close, but not equal to, *β*_0_, then

(10) $$W_{j}^{\text{opt}}(\beta )\approx \gamma^{1}\left(t_{(\;j),\beta };\beta \right)-\gamma^{0}\left(t_{(\;j),\beta },\beta \right)$$

Typically, *γ* ^0^(*t*;*β*) and *γ* ^1^(*t*;*β*) will be unknown, but they can be estimated from the observed data: *γ* ^*r*^(*t*;*β*) is estimated as the number of patients with *R* = *r*, $T_{0}(\beta )\geq t$ and *x*(*t*;*β*) = 1 divided by the number of patients with *R* = *r* and $T_{0}(\beta )\geq t$. We will refer to the weights defined in Equation (10) as ‘simple causal weights’.

### 3.6 Censored observations

For ease of explanation, we have so far assumed in this section that every subject's event time is observed, but real trial data will be inevitably be affected to some degree by censoring. Let *C*_*i*_ denote patient *i*'s (potential) censoring time. If *C*_*i*_<*T*_*i*_, then *T*_*i*_ is censored. For those with *C*_*i*_>*T*_*i*_, *C*_*i*_ should represent the end of planned follow up. In the context of an ITT analysis, provided censoring is non‐informative on the observed event timescale, that is *C*_*i*_⊥⊥*T*_*i*_, the type‐I error rate of the logrank test is asymptotically equal to its nominal value.

In a RPSFTM analysis, in order that the type‐I error rate is maintained for the null hypothesis $H_{0,\beta_{0}}$ (and so the nominal coverage of confidence intervals for *β* is maintained), we need to ensure that the censoring remains independent of failure times on the *β*‐transformed scale. This can be achieved in the following manner, as in 6. Let *Δ*_*i*_(0) = *I*(*T*_*i*_<*C*_*i*_) be a failure indicator on the original timescale denoting whether *T*_*i*_ is observed and assume that treatment switching is possible in both arms of the trial. Let *C*_*i*_(*β*) = $\min\{C_{i},C_{i}\exp(\beta )\}$ be patient *i*'s censoring time on the *β*–transformed scale. Their corresponding failure indicator on this time‐scale, *Δ*_*i*_(*β*), is given by

(11) $$\Delta_{i}(\beta )=\left\{\begin{array}{cc} 1 & \text{if}\;T_{0,i}(\beta )<C_{i}(\beta )\\ 0 & \text{otherwise}. \end{array}\right.$$

This scheme makes it possible for originally uncensored individuals to become censored on the *β*‐transformed scale but does not allow originally censored individuals to become uncensored. Thus, we define *J*(*β*) ( J(β)≤J) to be the number of unique event times on the *β*‐transformed timescale. To test *H*_0,*β*_, we re‐calculate logrank test statistic (1) using the *J*(*β*) ordered failure times on the *β*‐transformed timescale, *T*_(1),*β*_,…,*T*_(*J*(*β*)),*β*_.

## 4 Simulation study

In this section, we use a simulation study to compare the performances of the standard and weighted log‐rank test statistics: firstly, for testing the null hypothesis of no treatment effect in an ITT analysis (using the simple ITT weights from Equation (4) in Section 2.2) ; and secondly, for estimating the causal parameter *β* using a RPSFTM (using the simple causal weights from Equation (10) in Section 3.2). We do not consider the optimal weights defined in Equations (3) (for the ITT analysis) and (9) (for the causal RPSFTM analysis) any further. In a preliminary investigation, their performance was found to be highly similar to the simple weights, even when implemented in the most favourable circumstances (that is, by correctly plugging in the true values for either *θ*_0_ or *β*_0_). Section 4.1 describes our framework for simulating data using a RPSFTM. In Section 4.2, we specify four simulation scenarios by choosing which causal working assumptions to meet and which to violate. Results are presented in Sections 4.3 and 4.4.

### 4.1 Framework for data generating process

Patients randomised to the experimental arm (*R* = 1) start on the active treatment. If their disease progresses (‘progression 1’), they are switched to standard (control) therapy for the remainder of the trial. Patients randomised to the control arm (*R* = 0) start on standard therapy. If they experience a disease progression (also ‘progression 1’), they are then switched to the active drug. However, if they suffer a further progression (‘progression 2’), they are switched back to the control treatment for the remainder of the trial. Thus, regardless of the arm to which a patient is randomised to, there is a good chance that, before death, he/she will spend some time on and off the active treatment.

Time‐to‐event data following this treatment switching pattern were generated. As a first step, three independent random variables were simulated for each person *i* from the following exponential model:

(12) $$D_{\mathrm{ij}}∼\exp\left(\lambda_{\mathrm{ij}}^{-1}\right)\;j=1,2,3,\;\lambda_{\mathrm{ij}}=\lambda_{i}\exp\left(\mu_{j}+\eta_{i}\right),$$

where $\lambda_{\mathrm{ij}}^{-1}$ is the rate parameter (hence E[*D*_*ij*_] = *λ*_*ij*_). The ‘treatment–free’ times to disease progression ‘1’, disease progression ‘2’ and death are then given, respectively, by *D*_*i*1_, (*D*_*i*1_+*D*_*i*2_) and *D*_*i*3_. It is imagined that these times are on the month scale. The *μ*_*j*_ represent fixed parameters, whereas *λ*_*i*_ and *η*_*i*_ represent (potentially) random frailty terms for patient *i*. In all simulations *μ*_1_=*μ*_2_=*μ*_3_=2.5. Before discussing the assumed distributions of *λ*_*i*_ and *η*_*i*_ — and its implications for the distribution of *D*_*ij*_ — we describe the rest of the data generating process.

**Figure 2:**
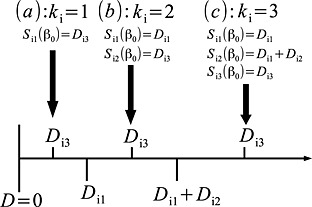
Illustration of the possible temporal orderings of person *i*'s treatment free events (switching and failure times). *D*_*i*3_ can occur before (position (a)), in between (position (b)) or after (position (c)) both disease progressions.

Whilst progression 2 can not occur before progression 1, death can occur before, in between or after the two progressions, as illustrated in Figure 2. Person *i*'s *D*_*i*1_, (*D*_*i*1_+*D*_*i*2_) and *D*_*i*3_ are temporally ordered and relabeled as either *S*_*i*,1_(*β*_0_), *S*_*i*,2_(*β*_0_) or *S*_*i*,3_(*β*_0_) according to the following principles: Death is the final event for person *i*, so events occurring after *D*_*i*3_ are subsequently ignored. Person *i*, therefore, has *k*_*i*_ pertinent potential events ( $S_{i,1}(\beta_{0}),\ldots ,S_{i,k_{i}}(\beta_{0})$), where *k*_*i*_∈{1,2,3}. For example, if *D*_*i*3_ is in position (c) then *k*_*i*_ = 3, *S*_*i*,1_(*β*_0_) = *D*_*i*1_, *S*_*i*,2_(*β*_0_) = *D*_*i*1_+*D*_*i*2_ and *S*_*i*,3_(*β*_0_) = *D*_*i*3_. Or, if *D*_*i*3_ is instead in position (a), then *k*_*i*_ = 1 and *S*_*i*,1_(*β*_0_) = *D*_*i*3_. Note that, for convenience, we are now using a common notation, *S*, to denote switching times *and* failure times, unlike in Section 3.2.

For a given value of the true causal treatment effect, *β*_0_, observed event times are then calculated using

(13) $$S_{i,j}=\overset{j}{\underset{l=1}{\sum }}(S_{i,l}(\beta_{0})-S_{i,l-1}(\beta_{0}))\exp\{-\beta_{0}X_{i,l}\},\;\text{for}j=1,\ldots ,k_{i}.$$

where *X*_*i*,*j*_ are the treatment indicator variables defined in Section 3 and *S*_*i*,0_=*S*_*i*,0_(*β*_0_) = 0 for all *i*. Note that Equation (13) is the inverse of Equation (7). The general treatment switching pattern observed over time in the two arms is

$$\begin{array}{cc} R & =1:\text{On}\to \text{Off}\\ R & =0:\text{Off}\to \text{On}\to \text{Off}, \end{array}$$

and so the implied full vector for (*X*_*i*,1_,*X*_*i*,2_,*X*_*i*,3_) is (1,0,0) for the *R* = 1 group and (0,1,0) for the *R* = 0 group, although only the first *k*_*i*_ entries of this vector are relevant to person *i*.

### 4.2 Data generation and our causal working assumptions

When we wish to satisfy the working assumptions used to derive the form of the simple causal weights, that is, CWA1 and CWA2, we set *λ*_*i*_=0.75 and *η*_*i*_=0 for all *i*. In that case, *D*_*ij*_ are i.i.d exponential with rate parameter $\lambda_{j}=\lambda \exp(\mu_{j})$. When we wish to violate these working assumptions, we set *λ*_*i*_∼ Uniform(0.6 and 0.9) and *η*_*i*_∼ N(0,1). The *D*_*ij*_'s are not then i.i.d exponential, because of the patient–specific frailty terms. This means that: (i) when the *D*_*i*,*j*_'s are transformed to *S*_*i*,*j*_(*β*_0_)'s, $S_{i,k_{i}}(\beta_{0})$ (which equals person *i*'s *T*_0,*i*_(*β*_0_)) is no longer i.i.d exponential, a violation of CWA2; (ii) when the *S*_*i*,*j*_(*β*_0_)'s are transformed to the *S*_*i*,*j*_'s via formula (13), the observed treatment switching times are correlated with the $S_{i,k_{i}}(\beta_{0})$'s (or *T*_0,*i*_(*β*_0_)'s), a violation of CWA1. In other words, the timing of a patient's treatment switches is predictive of their treatment—free failure time (i.e. their underlying prognosis). This in turn means that the hazard of a patient at observed time *t* will not only depend on *x*(*t*) but also on their full treatment history $\bar{x}(t)$, thus invalidating Equation (6).

In addition to generating the trial data as described earlier (from Equations (12) and (13)), we also considered two data–generating mechanisms that violated the BCA. Firstly, we assume that the treatment has a delayed effect of 3months, that is, a person's lifetime is used up at the ‘off treatment’ rate (1day per day) for the first 3months they take the treatment and thenceforth at rate exp(*β*_0_) until they stop treatment. They must, therefore, stay alive and on treatment for at least 3months to realise any potential benefit the treatment may hold. Secondly, we assume that the treatment's effectiveness is lessened or degraded after the first disease progression; specifically, the treatment effect is *β*_0_ before disease progression 1 and $\beta_{0}/\sqrt{2}$ afterwards.

To summarise, the four data generating mechanisms were as follows:

- Scenario 1: CWA1 & CWA2 true (*λ*_*i*_=0.75, *η*_*i*_=0), BCA true;
- Scenario 2: CWA1 & CWA2 false (*λ*_*i*_∼ Uniform(0.6,0.9), *η*_*i*_∼ N(0,1)), BCA true;
- Scenario 3: CWA1 & CWA2 false (*λ*_*i*_∼ Uniform(0.6,0.9), *η*_*i*_∼ N(0,1)), BCA false (3month delay to treatment effect), and
- Scenario 4: CWA1 & CWA2 false (*λ*_*i*_∼ Uniform(0.6,0.9), *η*_*i*_∼ N(0,1)), BCA false (1/ $\sqrt{2}$ degrading of treatment effect after progression 1).

**Figure 3:**
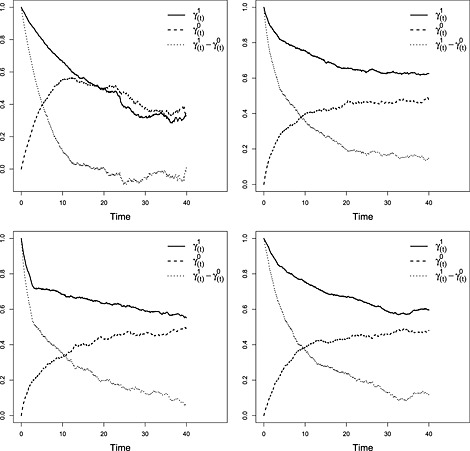
Treatment switching pattern for scenarios 1 (top‐left), 2 (top‐right),3 (bottom‐left),4 (bottom‐right), under *H*_1_, *β* = log(0.5). Results based on 20000 subjects.

**Figure 4:**
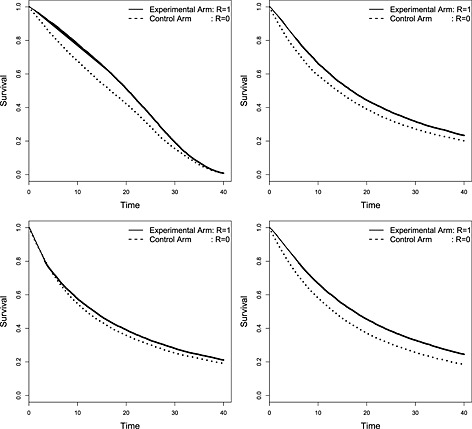
Kaplan Meier survival functions for scenarios 1 (top‐left), 2 (top‐right),3 (bottom‐left) and 4 (bottom‐right), under *H*_1_, *β* = log(0.5). Results based on 20000 subjects.

For each scenario, data were simulated both under *β*_0_ = 0 (so that the ITT null hypothesis *H*_0_ was true) and under $\beta_{0}\;=\;\log$(0.5) (so that *H*_0_ was false — this is referred to as *H*_1_). Two forms of right censoring were also introduced. For each subject, we generated a censoring time *C* from an exponential distribution with mean 250months. Additionally, everyone with a failure time above 40months (the assumed length of the trial follow up) was censored. This led on average to a censoring proportion of 10–15% across the distinct simulation scenarios. Figure 3 shows the basic pattern of treatment switching in the two groups across scenarios 1–4 under *H*_1_, and Figure 4 shows the Kaplan Meier survival curves for the two treatment groups across scenarios 1–4 under *H*_1_. These curves are shown for large trials of 20000 subjects, in order to reveal their true (large sample) shape.

### 4.3 Results: intention‐to‐treat analyses

**Figure 5:**
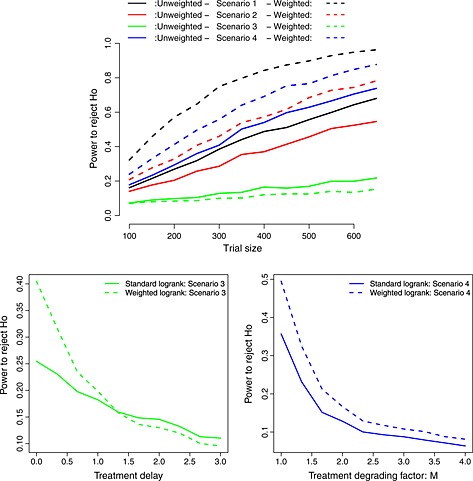
Top: Power to reject *H*_0_ under *H*_1_, *β* = log(0.5) for scenarios 1–4. Standard logrank test shown via solid lines; simple weighted logrank test shown via dashed lines. Bottom‐left: Power of the weighted and unweighted logrank test to reject *H*_0_ under *H*_1_ for scenario 3 and for a varying delay (trial size *n*= 250). Bottom‐right: Power of the weighted and unweighted logrank test to reject *H*_0_ under *H*_1_ for scenario 4 and for a varying degradation factor (trial size *n*= 250).

Table 1 (column 3) shows the type I error rate of the standard and simple weighted logrank test when used within an ITT analysis to test *H*_0_ at the two‐sided 5% significance level. All results in Table 1 are based on 5000 simulated trials of *n* = 250 patients. We see that, across all scenarios, the type I error rate remains close to the nominal level for both the standard and simple weighted ITT test. Figure 5 (top) shows the power to reject *H*_0_ under *H*_1_ using the standard and simple weighted ITT test, for scenarios 1–4 and for varying sample size (*n* = 100 to 650). Solid lines indicate power for the standard test, dashed lines indicate power for the weighted test, and scenarios are differentiated by colour. We see that the simple ITT weights furnish a substantially more powerful test in Scenarios 1, 2 and 4. To understand why this occurs, it is helpful to look at the relevant treatment switching patterns and Kaplan Meier curves in Figures 3 and 4, respectively. In each of these scenarios, large values of the weight function *γ* ^1^(*t*) − *γ* ^0^(*t*) coincide with a strong rate of separation in the Kaplan Meier curves during the first half of the trial (*t* = (0,20)). In Scenario 3, the standard logrank ITT test is more powerful than the simple weighted ITT test, although the power of both tests is fairly low. From Figure 4 (bottom‐left), we see that this is due to the delayed treatment effect preventing early separation of the survival curves, which is precisely when *γ* ^1^(*t*) − *γ* ^0^(*t*) is large. In order to better understand this result, we conduct a further simulation for a set of subcases of Scenario 3. Figure 5 (bottom‐left) shows the power of the standard and simple weighted ITT tests under *H*_1_ when the delay for treatment to take effect is varied between 0 and 3months. We see that the weighted ITT test is substantially more powerful for small delayed effects and remains more powerful than the standard test for up to a 1 $\frac{1}{2}$ month delay.

**Table 1:** Type I error of the standard and simple weighted ITT tests under *H*_0_ and bias/MSE of the standard and simple weighted RPSFTM analysis under *H*_1_ for scenarios 1–4.

Scenario	Logrank Method	ITT analysis	Causal RPSFTM analysis	
		*H*_0_: *β*_0_ = 0	*H*_1_: $\beta_{0}=\log(0.5)$	
		Type I error	Bias	MSE
1.	Standard	0.052	−0.003	0.204
	Simple Weighted	0.051	−0.009	0.089
2.	Standard	0.054	−0.029	0.277
	Simple Weighted	0.048	−0.037	0.170
3.	Standard	0.052	0.270	0.411
	Simple Weighted	0.051	0.417	0.381
4.	Standard	0.052	−0.117	0.266
	Simple Weighted	0.049	−0.094	0.176

ITT, intention‐to‐treatl; RPSFTM, rank‐preserving structural failure time model; MSE, mean squared error.

Figure 5 (bottom‐right) reports the results of a more in‐depth investigation of scenario 4. It shows the power of the standard and simple weighted ITT tests under *H*_1_ when the degrading factor is equal to 1/(M $\times \sqrt{2}$), for *M* in the interval from 1 to 4 (where *M* = 1 is Scenario 4). We see that the power of both tests reduces towards zero as M increases, but the weighted test always retains an advantage over the unweighted test.

### 4.4 Results: causal rank‐preserving structural failure time model analysis

Table 1 (columns 4 and 5) shows the bias and mean squared error (MSE) of estimates obtained for *β* within a RPSFTM analysis, using the standard (un‐weighted) logrank test and the logrank test with simple causal weights, for Scenarios 1–4. Unsurprisingly, both methods return unbiased estimates for the causal parameter in Scenario 1, but the MSE of the estimate obtained via the weighted analysis is only 44% of that obtained via an unweighted analysis. In Scenario 2, both tests returned estimates with a small amount of bias, with the weighted test being more severe. This bias was seen to diminish for larger trial sizes, however (results not shown). Again, MSE for the weighted analysis was considerably reduced compared with the standard unweighted analysis (approximately 61% of its magnitude).

Results for Scenarios 3 and 4 are more mixed, and harder to interpret, because the RPSFTM model is misspecified in both cases. For example, in Scenario 3 the treatment effect is (in truth) zero for 3months and then log(0.5) =−0.693 afterwards. Therefore, the ‘average’ treatment effect is somewhere between 0 and −0.693. The weighted logrank test estimates a treatment effect of around −0.28 (0.417 + log(0.5)) whereas the unweighted test's estimate is −0.42 (0.270 + log(0.5)). In Scenario 4, the bias is not so easily explained. The true effect is −0.693 before progression 1 and −0.693/ $\sqrt{2}$= −0.49 afterwards, and one might therefore expect the mean estimates to lie somewhere between these values. However, the mean estimates were approximately −0.81 and −0.79 for the unweighted and weighted approaches, respectively. By comparing the Kaplan Meier curves in Figure 4 (top‐right, no degradation) with Figure 4 (bottom‐right, degradation present), one can begin to see why the unweighted ITT logrank test statistic (and therefore the ITT hazard ratio) would be larger in Scenario 4 than in Scenario 2. In Scenario 4, the (*R* = 1) treatment group come off treatment at the point of disease progression 1 and do not take the treatment when its effect becomes degraded. The (*R* = 0) control group, by contrast, start treatment only after disease progression 1 has occurred, so only ever experience the degraded effect. The Kaplan Meier curve of the control group is not pulled towards that of the treatment group as quickly as it would have been had no degrading occurred (Scenario 2). Moving from Scenario 2 to Scenario 4 therefore reduces the benefit of post‐progression treatment for the placebo arm, thereby increasing the treatment effect estimated via an ITT or RPSFTM analysis.

In conclusion, hypothesis testing via an ITT analysis remains valid when the assumptions CWA1, CWA2 and BCA are violated, but the RPSFTM parameter will only be meaningful in general when the BCA is true. When the BCA is violated, the standard and weighted RPSFTM will identify different causal parameters depending on the treatment switching pattern and the type of violation.

## 5 Real data examples

In this section, we apply our weighted approach to re‐analyses of the Sunitinib trial 1, 13 and the Concorde trial 4, 8.

### 5.1 The Concorde trial

**Figure 6:**
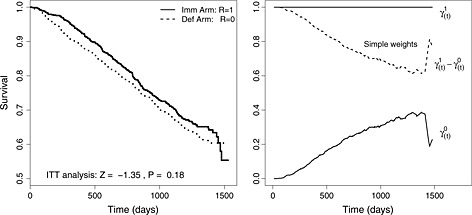
Concorde trial left: survival curves for the Imm and Def arms. Right: Proportion of people in the Imm and Def Concorde arms on treatment as a function of survival time.

Figure 6 (left) shows the Kaplan–Meier survival curves (for the earliest time to either AIDS, ARC or death) of the 1745 patients with HIV who were randomised to the Imm (*R* = 1) or Def (*R* = 0) arms of the Concorde trial. The logrank test statistic, for a comparison of the two survival curves, was −1.35, with an associated hazard ratio estimate of 0.89 and a *p*‐value of 0.18. It was subsequently questioned whether a departure from the original protocol, which enabled those in the Def arm to receive Zidovudine before a diagnosis of AIDS or ARC, had diluted the difference between the two treatment strategies. For this reason, White *et al*. 8 applied the RPSFTM (with the standard logrank test) to ask what would have happened if the original trial protocol had been followed with 100% adherence. To this end, they defined the treatment indicator variable for patient *i* at time *t*, *X*_*i*_(*t*), as:

$$X_{i}(t)=\left\{\begin{array}{ll} 1 & \text{if}\;R_{i}=1\;\text{or}\;(R_{i}=0\;\text{and}\;\mathrm{AZ}T_{i}\leq t)\\ 0 & \text{Otherwise}. \end{array}\right.$$

where *A* *Z* *T*_*i*_ was the time at which patient *i* initiated Zidovudine treatment. The causal parameter *β* is then defined using *X*_*i*_(*t*) via Equation (5). We aim to repeat their analysis using our simple weighted approach. Figure 6 (right) shows *γ* ^*r*^(*t*), (*l* = 0,1) for the Concorde trial.

The function *γ* ^1^(*t*) is fixed at 1 throughout; not because all patients in the Imm arm were on Zidovudine for their total participation in the trial (many would have stopped treatment earlier on their doctor's advice) but because all satisfied the original protocol of beginning Zidovudine treatment immediately, however briefly. Our simple weight from  Equation (4), for use within an ITT analysis, is therefore 1 − *γ* ^0^(*t*). A further consequence of *γ* ^1^(*t*) being fixed at 1 is that, in a RPSFTM analysis, no patient in the treatment arm who was originally uncensored can be ‘re‐censored’, regardless of the proposed value of *β*. This can be guaranteed by setting person *i*'s censoring indicator on the *β*–transformed scale, *C*_*i*_(*β*), equal to $C_{i}\exp(\beta )$(see 8 for further explanation). Before proceeding with an analysis of the Concorde trial, we first introduce our second real data example.

### 5.2 The Sunitinib trial

**Figure 7:**
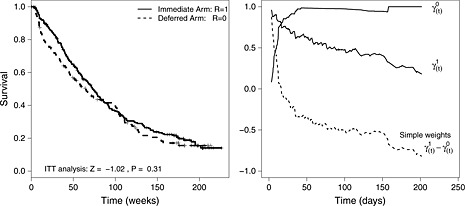
Sunitinib trial left: survival curves for the immediate and deferred arms. Bottom right: proportion of people in the immediate and deferred arms on treatment as a function of survival time.

Figure 7 (left) shows the Kaplan–Meier survival curves (time to death) for the 312 patients with gastrointestinal stromal tumours who were randomised to the Sunitinib arm (*R* = 1) and placebo arm (*R* = 0) as part of a multi‐centre phase III randomised controlled trial. The trial was unblinded approximately 50weeks after study initiation because of large observed differences in tumour progression rates between the two groups (in favour of Sunitinib).

At this point, patients in the placebo group were allowed to switch treatments and receive the new drug, which they did *en masse*. We label the two arms as ‘immediate’ or ‘deferred’ (Sunitinib) for convenience.

By the end of the trial, the highly significant difference in tumour progression rates was not mirrored in the overall survival measure between the two treatment groups. The final hazard ratio estimate reduced from 0.49 (*p*‐value 0.007) at the interim to 0.88 (*p*‐value 0.31). In order to investigate whether the disappearance of any treatment effect was due to the treatment switching, Demetri *et al*. 14 conducted a causal analysis using the RPSFTM with the standard logrank test, which was later discussed in 13. Here, we follow their example by defining the treatment indicator variable for patient *i* at time *t*, *X*_*i*_(*t*), as 1 if patient *i* was on Sunitinib at time *t* and 0 otherwise. This defines the parameter *β* as the causal effect that would have been observed if all patients on the immediate arm had received Sunitinib for the entire duration, and all patients on the deferred arm had received placebo

Figure 7 (right) shows the simple weight function for the Sunitinib trial. *γ* ^1^(*t*) decreased over the follow‐up period because a sizable proportion of patients in the immediate arm discontinued treatment at the point of tumour progression if their physician thought they would no longer benefit. *γ*_0_(*t*) is seen to increase rapidly from 0 to near 1 after 50weeks of follow up. The switching in the Sunitinib trial was so extreme that, from 25weeks of follow‐up onwards, the simple ITT weights *γ* ^1^(*t*) − *γ* ^0^(*t*) are negative. Some patients who were randomised to the control arm, and who switched over to Sunitinib, would have additionally come off treatment before death. Although our method could account for this pattern (indeed, the design of the simulation study did just that), this extra information was not available for these data. Our analysis therefore assumed that the treatment indicator variable for patients in the control group was fixed to 1 from the time they started to take Sunitinib to the end of follow‐up. It should therefore be treated as illustrative and conditional on this fact.

Under the assumptions used in their derivation, we would expect a negative weight at time *t* to coincide with a negative value for the difference between observed and expected numbers of events at time *t*, because the control group becomes the *de facto* treatment group. This would mean the numerator of the weighted logrank statistic in Equation (2) would still be positive, and the test would remain powerful. However, some may not be comfortable with this interpretation or the idea of negative weights at all. For this reason, we perform additional weighted ITT and causal analyses by truncating the simple weights in Equations (4) and (10) at 0 if they are negative. The truncated weights were also thought to provide a closer approximation to the true weights that would have been estimated and had full information on the treatment status of control group patients in the latter stages of the trial been available (as this would have probably have acted to restrict the weights from becoming negative in the first place).

### 5.3 Intention‐to‐treat and rank‐preserving structural failure time model analyses

**Figure 8:**
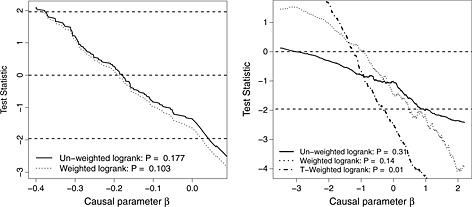
Estimation of the rank‐preserving structural failure time model causal parameter *β* using the standard and weighted logrank tests for the the Concorde trial (left) and the Sunitinib trial (right). ‘T‐weighted’ refers to the zero‐truncated weighted analysis.

An ITT analysis of the Concorde data using the simple weighted logrank test yielded a test statistic *Z* ^W^ of −1.64 with a corresponding *p*‐value of 0.10. Figure 8 (left) and Table 2 shows the results of a causal RPSFTM analysis using both the unweighted logrank test and the weighted test with the simple weights from Equation (10).

**Table 2:** ITT and RPSFTM causal analyses of the Sunitinib and Concorde trials using the standard and weighted logrank test. *Z* ^*w*∗^ and ${\hat{\beta }}^{*}$ refer to the test statistic and estimate, respectively, based on zero truncated weights (Sunitinib trial only).

Trial	Switching pattern		Analysis	Standard	Simple weighted
example	*R*=0	*R* = 1	type	logrank test	logrank test
			ITT	Z = −1.35 (0.177)	Z^*w*^ = −1.63 (0.103)
Concorde	Off→On	On			
			RPSFTM	$\hat{\beta }$ = −0.178 (−0.378, 0.0411)	$\hat{\beta }$ =‐0.188 (−0.386,0.0234)
			ITT	Z = −1.03 (0.306)	Z^*w*^ = −1.49 (0.137)
					Z^*w*∗^ = −2.48 (0.013)
Sunitinib	Off→On	On→Off			
			RPSFTM	$\hat{\beta }$ = −3.01 (−3.95, 0.88)	$\hat{\beta }$ = −0.89(−3.06,0.56)
					${\hat{\beta }}^{*}$ = −1.25(−2.21,−0.32)

ITT, intention‐to‐treatl; RPSFTM, rank‐preserving structural failure time model.

A 95% confidence interval for *β* under each test can be read off from Figure 8 as the range of *β* values for which test statistics lie between ± 1.96. The point estimate for *β* (where the test statistic equals 0) is slightly larger using the weighted test and its 95% confidence interval is around 2% narrower.

An ITT analysis of the Sunitinib data using the simple weighted logrank test yields a test statistic of −1.49 and a *p*‐value of 0.137. An ITT analysis using the zero‐truncated simple weights yields a test statistic of −2.48 with a *p*‐value of 0.013. Figure 8 (right) and Table 2 shows the results of a causal RPSFTM analysis using the unweighted logrank test, the simple weights from Equation (10) and the zero‐truncated simple weights.

There is considerable uncertainty as to the point estimate for *β* based on the standard and simple weighted tests — in both cases the test statistic is not a monotonically decreasing function of *β*, crossing zero several times. The simple weighted statistic yields a 95% confidence interval for *β*, which is approximately 43% narrower than that obtained using the standard logrank test. However, the zero‐truncated simple weights furnish the most precise estimate for *β* ( $\hat{\beta }$=−1.25, 95% C.I −2.21,−0.32).

## 6 Discussion

Given the severity of the disease, it is of paramount importance that clinical trials in late–stage cancer meet the needs of the patients who take part, as well as future patients. For this reason, the emphasis is usually, and rightly, on the pragmatic evaluation of different treatment strategies, rather than of simple all–or–nothing comparisons. Amendments to the original protocol are also therefore likely, if felt to be in the patients' interest. In this paper, we have shown that a simple weighted logrank test can be far more powerful than the standard logrank test for testing the ITT null hypothesis of no difference in the survival distributions between randomised groups when (a) substantial treatment switching occurs and (b) large rates of separation between the two group's survival functions over time coincides with large differences in the proportion of patients on treatment in each arm. This remained true even when the assumptions used to derive the weights were violated, and, when this was the case, the weighted test's type I error rate was maintained at its nominal level.

The evidence gathered from late‐stage cancer trials often needs to be subsequently interpreted by external bodies who are tasked with making decisions about a single treatment's suitability for future patient populations. This may require an assessment of its effectiveness compared with another therapy under different conditions than those originally followed in the trial. The RPSFTM has been used extensively to provide quantitative answers to such ‘what if’ questions. In addition to showing its use for ITT analyses, we have also shown that the weighted logrank test can furnish a more powerful method for comparing counterfactual treatment assignments within the RPSFTM framework, assuming that the assumptions of the RPSFTM hold.

It is important to stress that rejection of the null hypothesis by the weighted test should not be automatically equated with a conclusion of treatment superiority. For example, a reviewer highlighted the scenario (not explicitly explored in this paper) where being treated with an experimental drug at time t has the effect of reducing the individual's hazard at time t when *t* < *A*, but increasing it when *t* > *A*, for some time *A*. That is, the treatment causes benefit early on, but harm later. Suppose that this change in the direction of treatment effect causes the survival curves of the two treatment arms eventually to cross over. In this scenario, the early benefit and later harm of the treatment might cancel one another out in the test statistic of the standard unweighted log rank test, so that the null hypothesis of no treatment effect were not rejected. Any test statistic that gave more weight to earlier event times, on the other hand, might be more likely to reject the null hypothesis in favour of the experimental drug. In particular, this would be true of the weighted log rank test in the presence of treatment switching. In a very real sense, it is correct to reject the null, because the survival curves are truly different, and so the failure of the standard log rank test to reject the null constitutes a type‐II error 15, but it is important not to interpret this falsity of the null as being the same as treatment benefit.

If one believed that the treatment effect may not be constant over time, even in the absence of any treatment switching, then an extended RPSFTM could easily be used to probe this possibility via an additional sensitivity analysis. For example, to explore the case where the experimental treatment offered a reduced (or even negative) benefit over standard therapy after time *A*, then a two‐parameter RPSFTM of the form:

(14) $$T_{0}(\beta_{0},\psi_{0})=\exp\{\beta_{0}\}T_{\text{on}}^{\text{Pre–A}}+\exp\{\psi_{0}\}T_{\text{on}}^{\text{Post–A}}+T_{\text{off}},$$

could be fitted instead. Here, $T_{\text{on}}^{\text{Pre–A}}$ and $T_{\text{on}}^{\text{Post–A}}$ refer to time on treatment before and after time *A*, respectively, and the inclusion of a second parameter *ψ*_0_ allows for a different treatment effect either side of *A*. Time *A* could be common to all patients, or one could use patient—specific information such as time to disease progression. If Equation (14) is correctly specified, then *β*_0_ and *ψ*_0_ could be estimated consistently by g–estimation using this two–parameter RPSFTM. Similar models were investigated in the Concorde trial to explore the hypothesis that Zidovudine is more beneficial to patients with full‐blown AIDS than those who were only HIV‐positive 8. However, this was shown to be challenging because of the increased dimensionality of the parameter space. A simpler approach would be to define *ψ*_0_ (or perhaps even *ψ*_0_/*β*_0_) as a sensitivity parameter in Equation (14), and then find the values of *ψ*_0_ for which evidence of an apparent beneficial treatment effect would be brought into doubt.

Estimation of the RPSFTM causal parameter under the weighted logrank test is more of a computational challenge than when using the standard unweighted statistic. This is because each new proposal of *β* necessitates re‐censoring of the data, which in turn requires a re‐calculation of the weights. The parametric accelerated failure time modelling approach of Branson and Whitehead 16 uses a less stringent re‐censoring mechanism, and the additive hazard modelling approach of Martinussen 17 avoids the need for ‘artificial’ re‐censoring altogether. It would be interesting to see if these approaches could be extended to incorporate time‐varying treatment weights as outlined in this paper, to subsequently enable more precise causal estimates to be obtained with less computational effort.

## 7 Software

R code is available from the corresponding author to perform the analyses set out in this paper.
